# Goal consensus and task agreement as predictors of attendance and compliance in community-based treatments for adolescents with emotional disorders

**DOI:** 10.1186/s13034-025-00970-w

**Published:** 2025-11-20

**Authors:** Adam Panek, Emilie J. Butler, Amanda Jensen-Doss, Jill Ehrenreich-May, Golda S. Ginsburg

**Affiliations:** 1 Department of Psychiatry, University of Connecticut School of Medicine, 65 Kane Street Room 3022, West Hartford, CT 06119 USA; 2 Department of Psychology, University of Miami, P.O. Box 248185, Coral Gables, FL 33124-0751 USA

**Keywords:** Goal consensus, Task agreement, Emotional disorders, Community mental health, Attendance, Compliance

## Abstract

**Background:**

Several psychosocial treatments for adolescents with anxiety and depressive disorders have been shown to be effective. However, when evaluated in community clinics, response rates are poor, in part due to higher attrition and low treatment compliance. The working alliance between clinician and client is one important predictor of therapy attendance and compliance. Working alliance is comprised of three different components (i.e., goal consensus, task agreement, and bond). However, there is little research examining the unique contributions of two key components, goal consensus and task agreement. Exploring these components individually may shed light on important clinician-client interactions during therapy that might reduce attrition and improve compliance. The purpose of this study is to evaluate whether clinician and adolescent perceptions of agreement on (1) therapy goals (referred to as goal consensus) and (2) the tasks or actions needed to reach the goals (referred to as task agreement) predict therapy attendance and compliance in the context of a community-based randomized controlled trial of treatments for adolescents with emotional disorders.

**Methods:**

Participants include 89 clinicians (87.7% White) and 166 adolescents (mean age 14.7; 62.0% White) with a primary anxiety or depressive disorder enrolled in the Community Study of Outcome Monitoring for Emotional Disorders in Teens (COMET) study. Data on goal consensus and task agreement were collected digitally eight weeks after the initiation of treatment using the Working Alliance Inventory, reported by clinician and adolescent. Treatment compliance was rated by the clinician after each weekly session (1 = poor compliance to 7 = good compliance), and therapy attendance was measured by the total number of therapy sessions attended over 16 weeks.

**Results:**

Results of linear regression models, controlling for baseline anxiety/depression severity, treatment condition, and adolescent demographic characteristics, revealed that higher goal consensus (reported by both adolescent and clinician) significantly predicted higher attendance and compliance. Similarly, adolescent and clinician reports of higher task agreement predicted higher session attendance and treatment compliance.

**Conclusion:**

The current findings suggest that the more that adolescents and clinicians agreed on setting goals and the therapeutic tasks to achieve those goals, the more frequently adolescents attended therapy and complied with therapy expectations. Creating structured approaches to confirming goals and agreement on therapy tasks throughout treatment may be important and promote better treatment attendance and compliance and ultimately better outcomes for adolescents with emotional disorders.

## Introduction

Adolescent emotional disorders, such as anxiety and depression, are highly prevalent, with lifetime prevalence of anxiety disorders in adolescents as high as 32% and lifetime prevalence of mood disorders estimated to be 14.3% in the continental United States [15, 37]. Adolescent emotional disorders are associated with poor psychosocial outcomes, including lower life satisfaction, academic functioning, occupational functioning, family functioning, and social functioning [3, 46]. Rates of anxiety and depression in youth have risen substantially over the past several decades, leading to higher burden for adolescents and their families [3]. In fact, anxiety and depressive disorders are the two most burdensome mental health disorders in youth ages 15–19 in the United States [33]. Therefore, treating these disorders is imperative.

While there is considerable evidence supporting the efficacy of psychosocial treatments for emotional disorders, such as Cognitive Behavioral Therapy (CBT; [30]) or Interpersonal Therapy (IPT, [8]), when tested in outpatient academic efficacy trials, their effectiveness in community settings is lower [2, 50]. There are several potential reasons for this gap in effectiveness between research and practice settings [45]. For instance, patient characteristics in community settings are often more complex (e.g., greater comorbidity), patients are less likely to complete the recommended dosage on the intervention, and clinicians have poorer fidelity when implementing the treatments [17, 50].

Studies examining predictors of youth outcomes in community settings have found that family income [49], race, ethnicity [20], and baseline severity [39, 44] significantly impact response rates and thus are important covariates in future studies. Two understudied reasons for the lack of response in community settings are high dropout rates and low compliance with treatment expectations [27, 48]. Because research has demonstrated that both attendance and compliance are important considerations for improving therapy outcomes for youth [42], it is vital to understand factors that increase attendance and compliance.

### Working alliance

The working alliance, sometimes referred to as the therapeutic alliance, was defined by Bordin [6] as a relationship in which clinician and client engage collaboratively with a purpose. The working alliance has been established as one of the most important predictors of better treatment outcomes in adults [19], even when accounting for client characteristics, treatment approaches, and other variables (e.g., geographic location and virtual versus in-person). Studies examining common factors of various therapeutic approaches have shown that the working alliance between clinician and client is one of the most important treatment ingredients [1, 19, 43]

Research on the global construct of the working alliance among youth is more limited [11, 14, 41]. A recent review of the working alliance literature highlighted the need for further study of the working alliance components specifically in relation to pediatric anxiety disorders [9]. One study conducted by Hawley and Weisz [26] examined 65 youth ages 7–16 years that were receiving treatment from outpatient community mental health services. Youth and parent report of working alliance were examined in relation to patient satisfaction and therapy retention. Therapy retention was defined as the percentage of family attendance at the sessions, percentage of missed or canceled sessions, and the collective agreement that it was appropriate to terminate therapy. Results indicated that youth report of working alliance was not associated with higher therapy retention. However, parent report of working alliance predicted higher therapy attendance, fewer cancelations, and more frequent family participation. While this study highlights the importance of the working alliance and therapy attendance, the authors noted that future research should examine the relation between the working alliance and treatment compliance. Moreover, working alliance was measured as a global construct, yet it has several components, and these individual components were not considered.

Working alliance is comprised of three components, bond (the relationship between client and clinician), goal consensus (agreeing on therapy goals), and task agreement (agreeing on a set of therapy tasks to reach goals) [6]. While studies have demonstrated that the global construct of working alliance predicts treatment compliance or attendance in adolescent populations [12, 22], rarely have the components of the working alliance been examined independently, a noted underdeveloped area of the current working alliance literature [29]. The current literature on working alliance that does focus on individual components tends to examine the bond between clinician and their patient [13], and goal consensus and task agreement are comparatively understudied. For this reason, they are specifically examined in the current study as they might shed light on other important dimensions of working alliance.

### Goal consensus

One of the three individual working alliance components, goal consensus (also referred to in the literature as goal agreement or goal alliance), is defined as the collaborative therapy goal setting process between client and clinician [6]. While few studies have explored goal consensus specifically, a growing literature has examined related and overlapping constructs. For example, a recent review found that goal setting and shared decision-making promote therapy attendance in youth with emotional disorders [48]. Research in youth community mental health settings has shown that goal-centered constructs have a relationship with attendance and compliance [52]. For instance, Cairns et al. [10] examined goal setting (i.e., whether therapy goals were set) in relation to attendance in general youth community mental health services. Within a sample of 283 Australian youth ages 12–25, goal setting was associated with a higher number of therapy sessions attended. However, despite these encouraging findings, only 66% of the sample reported that goals were set as part of their therapy, which suggests that within community mental health settings, there may be inconsistency regarding whether goal setting is incorporated into therapy. One limitation noted by Cairns and colleagues is that goal setting was measured as a dichotomous variable (yes/no), and did not capture whether goals were negotiated, discussed, or a consensus was reached. This signifies a strong need for research that highlights both clinician and youth perspectives to confirm whether they *agree* with the goals that have been set and the steps necessary to get there.

### Task agreement

Another understudied core component of the working alliance that may be relevant to attendance and compliance is task agreement (sometimes referred to as task alliance), which is defined as agreement on the specific steps (i.e., tasks) that the client agrees to complete to reach their agreed-upon goals [6]. Few studies have examined the specific role of task agreement in relation to attendance or compliance [29], and to date, this relationship has not been tested in adolescent populations. However, insights can be taken from research examining working alliance components in adult populations. For example, Hagen et al. [24] investigated the link between task agreement via client and clinician report (measured by the Working Alliance Inventory—Short; [47]) and treatment outcomes (measured by the Yale-Brown Obsessive Compulsive Scale; [21]) from 44 outpatients with obsessive–compulsive disorder undergoing exposure and response prevention treatment. Results indicated that clinician (but not client) report of task agreement predicted better treatment outcomes. However, this relationship has not been examined in adolescent samples, indicating the need for further research.

### Current study

As adolescent emotional disorders are highly prevalent and burdensome on both individual and systemic levels, there is a pressing need to provide intervention, especially in community settings where access to care and treatment engagement and outcomes are lower. Therefore, the current study assessed goal consensus and task agreement as predictors of attendance and compliance in adolescents with emotional disorders (anxiety and depression). Specifically, we examined whether adolescent and clinician reports of goal consensus and task agreement were associated with therapy attendance and clinician-rated compliance, in the context of a community-based randomized controlled effectiveness trial of treatments for adolescents with emotional disorders. Data from the National Institute of Mental Health (NIMH)-funded Community Study of Outcome Monitoring for Emotional Disorders in Teens (COMET) study [31] were leveraged to examine the current aims. COMET was a randomized controlled trial of three treatment conditions: Treatment as usual (TAU), a standardized feedback questionnaire (Youth Outcomes Questionnaire, YOQ) with treatment as usual (TAU+), and a transdiagnostic treatment method for emotional disorders (the Unified Protocol for Transdiagnostic Treatment of Emotional Disorders in Adolescents, UP-A) including the YOQ. For the purposes of this study, the conditions were not evaluated separately to determine the role of goal consensus and task agreement regardless of treatment model, as these constructs are considered common factors across therapies. It was hypothesized that higher goal consensus and task agreement, reported by both adolescent and clinician, would predict higher therapy attendance and higher clinician-rated treatment compliance.

## Methods

### Participants

#### Clinicians

Eighty-nine clinicians participated (*M* age = 35.5, 69.7% White, 87.7% Female) in the current study. All clinicians were at least part time employees, trainees, or completed an approved practicum or internship of at least one year in participating clinics, and were able to speak, read, and understand English. The clinicians that were included in the current analysis completed at least one session with an adolescent. Clinician characteristics are detailed in Table 1.

**Table 1:** Clinician characteristics (N = 89)

Characteristic	*N*
Females	78 (87.7%)
Males	11 (12.4%)
Mean age (standard deviation)	35.5 (10.1)
Ethnicity (Hispanic/Latino % Yes)	28 (31.5%)
Race % yes (white)	62 (69.7%)
(Black)	19 (21.3%)
(Other)	8 (9.0%)
Highest earned degree (% with Masters)	82 (92.1%)
% with doctorates	4 (4.5%)
% with bachelor’s	3 (3.4%)
Total years in current position	2.5 (4.0)
Licensed to perform mental health services (% yes)	28 (32%)

The racial category of “Other” includes Asian, Native Hawaiian/Pacific Islander, and those who reported being of mixed race

#### Adolescents

Two hundred and twenty-two adolescents between the ages of 12 and 18 with clinically significant symptoms of anxiety or depression and their parents (79.2% mothers) were enrolled in the COMET study [31]. Inclusion criteria included clinically significant symptoms of any anxiety and/or depressive disorders (as assessed by independent evaluators using the Anxiety Disorders Interview Schedule for the DSM-5, Child and Parent Report [ADIS; [4]), living with a legal guardian that could attend treatment sessions at least half of the time, and having a caregiver capable of completing study procedures in English or Spanish. Exclusion criteria consisted of receiving current psychosocial interventions, suicidal behavior that required more care than an outpatient setting, and any factors that would make any outpatient treatment for emotional disorders inappropriate (i.e., significant substance abuse, IQ below 80). Further information about attrition rates and number of excluded adolescents can be found in the primary outcomes manuscript consort diagram from Ehrenreich-May et al. [17]. The current study included data from a sub-sample of 166 participants (*M* age = 14.7, 62.0% White, 33.1% Male) that attended at least one session with a clinician. Baseline characteristics of adolescents whose data were included in the current study are detailed in Table 2.

**Table 2:** Adolescent characteristics (N = 166)

Characteristic	*N*
Females	108 (65.1%)
Males	55 (33.1%)
Transgender (female to male)	2 (1.2%)
Other	1 (0.6%)
Mean age (standard deviation)	14.7 (1.7)
Ethnicity (Hispanic/Latino % yes)	57 (34.3%)
Race % yes (white)	103 (62.0%)
(Black)	37 (22.3%)
(Other)	26 (15.7%)
Family annual income	
5,000–19,999	32 (20.1%)
20,000–59,999	61 (38.3%)
60,000–99,999	36 (22.6%)
100,000 or over	30 (18.9%)
Primary diagnosis (%)	
Generalized anxiety disorder	73 (44.0%)
Social anxiety disorder	39 (23.5%)
Specific phobia	2 (1.2%)
Obsessive–compulsive disorder	6 (3.6%)
Post-traumatic stress disorder	2 (1.2%)
Major depressive disorder	17 (10.2%)
Persistent depressive disorder	16 (9.6%)
Unspecified depressive disorder	3 (1.8%)
Adjustment disorder with depressed mood	1 (0.6%)
Disruptive mood dysregulation disorder	1 (0.6%)
Attention deficit hyperactivity disorder	3 (1.8%)
Adjustment disorder with mixed anxiety and depressed mood	1 (0.6%)
% on psychiatric medication	40 (24.1%)

Only three adolescents identified as other than cisgender and were categorized with cisgender females in the analyses. The racial category “Other” included American Indian/Alaskan Native, Asian, Native Hawaiian/Pacific Islander, or those who identified as mixed race

### Procedure

Data from the current study was collected as a part of the NIMH-funded Community Study of Outcome Monitoring for Emotional Disorders in Teens (COMET) study which included multiple participating community mental health clinics from Florida and Connecticut [17, 31]. Clinicians were randomized to administer one of the three study treatments (UP-A, TAU, or TAU+) after completing baseline assessments and consent. Specifically, within every participating agency, blocks of three clinicians were randomly assigned using a random number generator (www.randomization.com). Clinicians were randomized within a separate block if they belonged to an agency with three clinicians who spoke Spanish. Following their randomization to the experimental conditions, clinicians received UP-A and/or TAU+ training and consultation, and all clinicians treated study cases as part of their normal caseload. The UP-A is a modular approach and includes the module Building and Keeping motivation. This includes identification of top problems and goals for treatment and motivational enhancement. TAU and TAU+ clinicians used the treatment modalities as they normally would, although they reported using cognitive or cognitive-behavioral elements (70% and 76% respectively; [17]). Clinicians completed a session summary form (after receiving training) after each visit to assess compliance. Measures of goal consensus and task agreement were collected digitally at the eight and 16-week points for each study therapy case. Clinicians received payment for their training and consultation hours either through their regular agency processes or directly from the study, depending on the organization.

Participant families were informed about the study during the first phone conversation with the agency or after completing the clinic's intake assessment; specific protocols differed clinic-to-clinic. Interested adolescents and their caregivers signed assent and/or consent forms (depending on age of the adolescent) and participated in a baseline study assessment by independent evaluators if they were eligible for services at the clinic. Independent evaluators held a master’s or doctorate degree or were enrolled in a doctoral program related to a child mental health field. Independent evaluator training included didactics, review by a more senior IE, and matching on co-rated diagnostic interviews on sample cases. (Further information on independent evaluator training can be found in [31]. Eligible adolescents were randomized to one of the three study conditions. To ensure that the sample of adolescents was evenly distributed regarding baseline characteristics, randomization was blocked on presence of depression, any psychiatric medication use, and English or Spanish language. Participant families completed a baseline semi-structured diagnostic interview, the ADIS, with independent evaluators, either in person or virtually, and completed online study questionnaires.

### Measures

#### Clinical global impression-severity (CGI-S)

The CGI-S is a widely used measure of symptom severity [23, 34]. It was used in the current study to assess overall anxiety and depression symptom severity on a seven-point scale. It ranges from 1 (No illness) to 7 (Extremely Ill) and was administered at each time point by an independent evaluator after completing the ADIS, to assess change in overall anxiety/depression severity. The CGI-S in the full sample demonstrated good interrater reliability (*ICC* = 0.80). In the current study, the CGI-S was used to control for baseline anxiety/depression symptom severity. The average impairment at baseline was 5.64 (*SD* = 0.78) and ranged from 3 to 7.

#### Demographic form

Adolescent characteristics such as race, gender, ethnicity, and family income were collected through adolescent and caregiver report. Gender included item responses of Male, Female, Transgender (Male to Female), Transgender (Female to Male), and Other.

#### Working alliance inventory: short form (WAI), goal consensus and task agreement subscales

Both adolescents and clinicians completed the Working Alliance Inventory, short form versions [25], which are twelve and ten items respectively, eight weeks after the adolescent started treatment. The short form versions have been cross validated with Bordin’s working alliance [6] and other alliance measures [25]. The Working Alliance Inventory has been commonly used in the child literature and has been well validated [32] Each item on both adolescent-report and clinician-report versions was rated on a five-point Likert scale from 1 (seldom) to 5 (always). The two scales of the Working Alliance used in the current study were goal consensus and task agreement. Adolescent report of goal consensus included four items, such as “___ and I collaborate on setting goals for my therapy” and “___ and I are working towards mutually agreed upon goals”. Adolescent ratings of goal consensus ranged from 1 to 5 (*M* = 3.58, *SD* = 1.09). The clinician report of goal consensus contained three items, including “We are working towards mutually agreed upon goals” and “___and I have a common perception of his/her goals”. Clinician ratings of goal consensus ranged from 1 to 5 (*M* = 4.12, *SD* = 0.96). The Cronbach’s alpha for goal consensus for adolescent report was 0.93 and was 0.89 for clinician report. Higher scores on the goal consensus subscale indicate higher perceived level of agreement between clinician and adolescent.

Regarding the task agreement subscale, the adolescent report consisted of 4 items, such as, “As a result of these sessions I am clearer as to how I might be able to change” and “I believe the way we are working with my problem is correct.” Adolescent report ratings again ranged from 1 to 5 (*M* = 3.76, *SD* = 1.06). For the clinician report, the task agreement subscale contained three items, such as “__and I agree about the steps to be taken to improve his/her situation” and “___and I both feel confident about the usefulness of our current activity in therapy”. Clinician report ratings ranged from 1 to 5 (*M* = 3.82, *SD* = 0.92). The Cronbach’s alpha for task agreement was 0.90 for the adolescent report and 0.87 for the clinician report. Higher scores on the task agreement subscale reflect higher perceived agreement between adolescent and clinician regarding the tasks or steps needed to achieve therapy goals.

#### Session summary form: treatment compliance and session attendance

After each therapy session with an adolescent, clinicians completed a post-session online form that included the item, “This question is about overall compliance with therapy, defined as how well the child completed the requirements of the therapy and followed your instructions,” which they rated from 1 (Poor compliance) to 7 (Good compliance). Compliance scores from each session were averaged into a mean score (the possible range was 1–7). The mean score in the current sample for compliance was 5.15 (*SD* = 1.15). Higher scores indicate better therapy compliance.

Additionally, clinicians indicated on the post-session form whether the adolescent attended each scheduled session, which was used to calculate a session attendance summary score from the baseline evaluation until the 16-week study timepoint. Sessions attended ranged from 1 to 16 and the average sessions attended were 9.33 (*SD* = 4.10).

## Data analysis

A total of eight separate hierarchical linear regression models with clinician and adolescent report of goal consensus and task agreement as predictor variables, and therapy attendance and clinician-report of therapy compliance as outcomes were employed to evaluate the study aims. To address the risk of a type 1 error, a Holms–Bonferroni correction was applied to the hierarchal linear regressions. The Holm–Bonferroni method is a sequentially rejective multiple testing procedure that controls the family-wise error rate more effectively than the traditional Bonferroni correction [28]. By adjusting the significance thresholds for each test, this method reduces the likelihood of false positives, ensuring that the results are robust and reliable. Treatment condition, baseline anxiety and depression severity, family income, adolescent gender, age, and race and ethnicity were included as covariates in the analyses. Categorical covariates were dichotomized to be used in regression models, however, this approach risks reducing statistical power and obscuring variability within groups. For race/ethnicity, non-Hispanic White was used as the reference category and coded as 1, other variables = 0. For the gender variable, male was the reference category and coded as 1 and female/other was coded as 0. The Statistical Product and Service Solutions 29 (SPSS) was used to analyze the data. Item-level missingness was addressed for the Working Alliance Inventory subscales (goal consensus and task agreement) by using a mean substitution if at least 70.0% of the data was present. The compliance item on the session summary form was calculated with available data for each case (i.e., for a participant with the compliance item rated for four sessions, an average across those four scores was calculated). Missing data ranged from 14.5% to 27.1% depending on the measure.

## Post hoc analysis

Due to high level of correlations between independent variables in the current analyses (see Table 3), the analytic plan involved running eight individual regression models to examine the current aims. However, even with the inclusion of Holms-Bonferroni corrections, the risk of Type 1 error (false positive results) remained a concern. Therefore, post-hoc analyses examined the relationships between variables of interest using fewer regression models. Clinician report and adolescent report of independent variables could not be included in the same regression models due to the risk of clinician effects, as clinicians in the current sample worked with multiple adolescents in the sample. As such, four regression models were run, with clinician report (task agreement and goal consensus) and adolescent report (task agreement and goal consensus) examined separately as predictors of the two outcome variables (number of therapy sessions and therapy compliance). The same control variables noted above were also included in these models.

**Table 3:** Intercorrelations among study variables

	Attendance	Compliance	CGI-S	Adolescent gender	Family income	Adolescent age	Race/ethnicity	TAU	TAU+	Child report—GC	Child report—TA	Clinician report—TA	Clinician report—GC
Attendance	1	0.181*	0.150	0.009	− 0.011	− 0.079	− 0.085	− 0.013	0.100	0.350**	0.348**	0.168*	0.174*
Compliance	−	1	− 0.153*	− 0.179*	0.207**	0.239**	0.076	0.058	0.300**	0.299**	0.217*	0.615**	0.543**
CGI-S	−	−	1	− 0.084	− 0.107	0.081	− 0.054	− 0.023	− 0.031	− 0.104	− 0.025	−0.112	− 0.026
Adolescent gender	−	−	−	1	− 0.113	0.042	− 0.117	0.084	− 0.043	− 0.175*	− 0.069	−0.085	− 0.086
Family income	−	−	−	−	1	0.039	0.350**	0.137	0.080	0.043	0.004	0.184*	0.091
Adolescent age	−	−	−	−	−	1	− 0.073	− 0.095	0.098	− 0.166	− 0.203*	0.094	0.081
Race/ethnicity	−	−	−	−	−	−	1	0.031	− 0.073	− 0.102	− 0.065	0.051	0.017
TAU	−	−	−	−	−	−	−	1	− 0.495**	− 0.084	− 0.017	0.144	0.087
TAU+	−	−	−	−	−	−	−	−	1	0.098	0.052	0.046	0.037
Child report—GC	−	−	−	−	−	−	−	−	−	1	0.861**	0.197*	0.171*
Child report—TA	−	−	−	−	−	−	−	−	−	−	1	0.163	0.149
Clinician report—TA	−	−	−	−	−	−	−	−	−	−	−	1	0.858**
Clinician report—GC	−	−	−	−	−	−	−	−	−	−	−	−	1

GC = Goal Consensus, TA = Task Agreement

*Correlation is significant at the 0.05 level (2-tailed)

**Correlation is significant at the 0.01 level (2-tailed)

## Results

Preliminary analyses revealed no differences across treatment conditions on the goal consensus or task agreement subscales (see Table 4 for mean goal consensus and task agreement scores by treatment condition). Mean sessions attended in the UP-A (UP-A *M* = 8.964, *SD* = 4.208) TAU, (*M* = 9.368, *SD* = 3.735) and TAU + (*M* = 10.019, *SD* = 3.925) conditions were not statistically different. Mean compliance was also not statistically different across treatment conditions: UP-A (*M* = 4.583, *SD* = 1.271), TAU (*M* = 5.245, *SD* = 0.968) and TAU + (*M* = 5.654, *SD* = 0.921). Finally, the magnitude of correlations between adolescent and clinician goal consensus (*r* = 0.265, *p* = 0.005) and task agreement (*r* = 0.242, *p* = 0.010) were low but statistically significant.

**Table 4:** Treatment condition mean by goal consensus and task agreement

	TAU (*N* = 57)	TAU + (*N* = 53)	UP-A (*N* = 56)
	Mean (SD)	Mean (SD)	Mean (SD)
Goal consensus—clinician report	4.183 (0.761)	4.203 (1.002)	3.954 (1.112)
Task agreement—clinician report	3.928 (0.770)	3.943 (0.922)	3.581 (1.040)
Goal consensus—adolescent report	3.968 (0.898)	4.094 (1.000)	3.965 (1.355)
Task agreement—adolescent report	3.702 (0.935)	3.783 (1.003)	3.792 (1.247)

TAU = Treatment as usual, TAU+ = Treatment as usual plus measurement-based care, UP-A = Unified protocol for transdiagnostic treatment of emotional disorders in adolescents and measurement-based care

### Goal consensus

The overall regression model for adolescent report of goal consensus predicting therapy attendance was statistically significant (*F* [1, 122] = 2.972, *p* = 0.004), as was the individual adolescent report variable (*B* = 1.392, *SE* = 0.322, *p* < 0.001), which accounted 12.8% (*R*^2^) of the variance in number of sessions attended. Full results can be found in Table 5.

**Table 5:** Regression analysis of goal consensus—adolescent report on therapy attendance

Predictors	Unstandardized coefficients		Standardized coefficients	95.0% confidence interval for B		t	Sig
	B	Std. error	Beta	Lower bound	Upper bound		
(Constant)	0.258	4.064		− 7.788	8.303	0.063	0.950
CGI-S	0.832	0.407	0.178	0.026	1.637	2.043	0.043
Adolescent gender	0.681	0.677	0.086	− 0.660	2.022	1.005	0.317
Family income	0.201	0.455	0.042	− 0.700	1.101	0.441	0.660
Adolescent age	− 0.096	0.193	− 0.043	− 0.477	0.286	− 0.496	0.621
Adolescent race/ethnicity	− 0.093	0.731	− 0.012	− 1.539	1.353	− 0.127	0.899
TAU	0.373	0.779	0.047	− 1.169	1.916	0.479	0.633
TAU +	0.536	0.778	0.067	− 1.005	2.077	0.688	0.492
Goal consensus—adolescent report	1.392	0.322	0.383	0.754	2.029	4.323	< 0.001*

CGI-S = Clinical Global Impression-Severity scale, TAU = Treatment as usual, TAU+ = Treatment as usual plus measurement-based care. Covariates in the model were race/ethnicity, family income, gender, age, and treatment condition. For race/ethnicity, non-Hispanic White was used as the reference category and coded as 1, other variables = 0. For the gender variable, male was the reference category and coded as 1 and female/other was coded as 0. Holms corrected *p < 0.006

The overall model using clinician report of goal consensus as a predictor of therapy attendance was not statistically significant (*F* [1, 142] = 1.156, *p* = 0.330), but the individual clinician report of goal consensus variable (*B* = 0.778, *SE* = 0.343, *p* = 0.025) was a statistically significant predictor of higher session attendance. Further, clinician reported goal consensus explained 3.4% (*R*^2^) of variance in number of sessions attended. More information can be found in Table 6.

**Table 6:** Regression analysis of goal consensus—clinician report on therapy attendance

Predictors	Unstandardized coefficients		Standardized coefficients	95.0% confidence interval for B		t	Sig
	B	Std. error	Beta	Lower bound	Upper bound		
(Constant)	6.597	3.645		− 0.608	13.802	1.810	0.072
CGI-S	0.383	0.397	0.080	− 0.402	1.168	0.964	0.337
Adolescent gender	0.468	0.667	0.058	− 0.850	1.786	0.702	0.484
Family income	− 0.029	0.444	− 0.006	− 0.907	0.849	− 0.065	0.948
Adolescent age	− 0.147	0.184	− .0.066	− 0.511	0.217	− 0.799	0.426
Adolescent race/ethnicity	− 0.474	0.705	− 0.059	− 1.869	0.920	− 0.673	0.502
TAU	− 0.505	0.773	− 0.064	− 2.033	1.023	− 0.654	0.514
TAU+	0.685	0.774	0.085	− 0.846	2.215	0.884	0.378
Goal consensus—clinician report	0.778	0.343	0.188	0.100	1.457	2.267	0.025*

CGI-S = Clinical Global Impression-Severity scale, TAU = Treatment as usual, TAU+ = Treatment as usual plus measurement-based care. Covariates in the model were race/ethnicity, family income, gender, age, and treatment condition. For race/ethnicity, non-Hispanic White was used as the reference category and coded as 1, other variables = 0. For the gender variable, male was the reference category and coded as 1 and female/other was coded as 0. Holms corrected *p < 0.05

The model examining adolescent-reported goal consensus as a predictor of therapy compliance was statistically significant (*F* [1, 121] = 7.525, *p* < 0.001), and the individual adolescent reported goal consensus variable emerged as a significant predictor (*B* = 0.359, *SE* = 0.084, *p* < 0.001) and explained 9.4% (*R*^2^) of the variance in therapy compliance (see Table 7).

**Table 7:** Regression analysis of goal consensus—adolescent report on clinician rated compliance

Predictors	Unstandardized coefficients		Standardized coefficients	95.0% confidence interval for B		*t*	Sig
	B	Std. error	Beta	Lower bound	Upper bound		
(Constant)	0.886	1.059		− 1.211	2.982	0.836	0.405
CGI-S	− 0.123	0.106	− 0.088	− 0.332	0.087	− 1.158	0.249
Adolescent gender	− 0.358	0.177	− 0.151	− 0.709	− 0.007	− 2.019	0.046
Family income	0.142	0.118	0.099	− 0.092	0.377	1.201	0.232
Adolescent Age	0.191	0.050	0.286	0.091	0.290	3.786	< 0.001*
Adolescent race/ethnicity	0.317	0.192	0.132	− 0.063	0.698	1.652	0.101
TAU	0.749	0.203	0.315	0.348	1.151	3.695	< 0.001*
TAU+	0.853	0.204	0.356	0.448	1.257	4.171	< 0.001*
Goal consensus—adolescent report	0.359	0.084	0.326	0.192	0.527	4.252	< 0.001*

CGI-S = Clinical Global Impression-Severity scale, TAU = Treatment as usual, TAU + = Treatment as usual plus measurement-based care. Covariates in the model were race/ethnicity, family income, gender, age, and treatment condition. For race/ethnicity, non-Hispanic White was used as the reference category and coded as 1, other variables = 0. For the gender variable, male was the reference category and coded as 1 and female/other was coded as 0. Holms corrected *p < 0.007

The overall model testing clinician report of goal consensus on therapy compliance was statistically significant, (*F* [1, 142] = 18.463, *p* < 0.001), and the individual variable of clinician reported goal consensus was also a significant predictor of increased therapy compliance (*B* = 0.589, *SE* = 0.077, *p* < 0.001). Clinician report of goal consensus explained 20.4% (*R*^2^) of the variance in therapy compliance (see Table 8).

**Table 8:** Regression analysis of goal consensus—clinician report on clinician rated compliance

Predictors	Unstandardized coefficients		Standardized coefficients	95.0% Confidence interval for B		*t*	Sig
	B	Std. error	Beta	Lower bound	Upper bound		
(Constant)	1.408	0.814		− 0.201	3.018	1.729	0.086
CGI-S	− 0.208	0.089	− 0.141	− 0.384	− 0.033	− 2.346	0.020
Adolescent Gender	− 0.462	0.149	− 0.186	− 0.757	− 0.168	− 3.103	0.002*
Family income	0.097	0.099	0.063	− 0.099	0.293	0.975	0.331
Adolescent age	0.134	0.041	0.196	0.053	0.216	3.272	0.001*
Adolescent race/ethnicity	0.218	0.158	0.087	− 0.094	0.529	1.383	0.169
TAU	0.554	0.173	0.226	0.212	0.895	3.207	0.002*
TAU+	0.957	0.173	0.386	0.616	1.299	5.535	< 0.001*
Goal consensus—clinician report	0.589	0.077	0.460	0.438	0.741	7.684	< 0.001*

CGI-S = Clinical Global Impression-Severity scale, TAU = Treatment as usual, TAU+ = Treatment as usual plus measurement-based care. Covariates in the model were race/ethnicity, family income, gender, age, and treatment condition. For race/ethnicity, non-Hispanic White was used as the reference category and coded as 1, other variables = 0. For the gender variable, male was the reference category and coded as 1 and female/other was coded as 0. Holms corrected *p < 0.008

### Task agreement

The overall regression model for adolescent report of task agreement on therapy attendance was statistically significant (*F* [1, 122] = 2.473, *p* = 0.016), and the individual adolescent report variable was also a significant predictor (*B* = 1.279, *SE* = 0.332, *p* < 0.001). Adolescent-reported task agreement accounted for 10.5% (*R*^2^) of the variance in number of sessions attended. Full results can be found in Table 9.

**Table 9:** Regression analysis of task agreement—adolescent report on therapy attendance

Predictors	Unstandardized coefficients		Standardized coefficients	95.0% confidence interval for B		t	Sig
	B	Std. error	Beta	Lower bound	Upper bound		
(Constant)	2.456	3.953		− 5.371	10.282	0.621	0.536
CGI-S	0.641	0.410	0.137	− 0.170	1.452	1.565	0.120
Adolescent gender	0.297	0.675	0.038	− 1.040	1.633	0.439	0.661
Family income	0.290	0.460	0.060	− 0.620	1.201	0.631	0.529
Adolescent age	− 0.102	0.196	− 0.046	− 0.489	0.286	− 0.519	0.605
Adolescent race/ethnicity	− 0.370	0.733	− 0.046	− 1.820	1.081	− 0.505	0.615
TAU	0.115	0.787	0.014	− 1.443	1.673	0.146	0.884
TAU+	0.493	0.790	0.062	− 1.070	2.056	0.624	0.534
Task agreement—adolescent report	1.279	0.332	0.334	0.622	1.936	3.852	< 0.001*

CGI-S = Clinical Global Impression-Severity scale, TAU = Treatment as usual, TAU+ = Treatment as usual plus measurement-based care. Covariates in the model were race/ethnicity, family income, gender, age, and treatment condition. For race/ethnicity, non-Hispanic White was used as the reference category and coded as 1, other variables = 0. For the gender variable, male was the reference category and coded as 1 and female/other was coded as 0. Holms corrected *p < 0.01

The overall model testing clinician report of task agreement as a predictor of therapy attendance was not statistically significant (*F* [1, 142] = 1.375, *p* = 2.12), but the individual clinician report of task agreement variable was a statistically significant predictor of higher session attendance (*B* = 0.915, *SE* = 0.350, *p* = 0.010). Clinician-reported task agreement explained 4.5% (*R*^2^) of the variance in number of sessions attended. More information can be found in Table 10.

**Table 10:** Regression analysis of task agreement—clinician report on therapy attendance

Predictors	Unstandardized coefficients		Standardized coefficients	95.0% Confidence interval for B		t	Sig
	B	Std. error	Beta	Lower bound	Upper bound		
(Constant)	6.317	3.611		− 0.821	13.454	1.749	0.082
CGI-S	0.460	0.397	0.096	− 0.325	1.244	1.158	0.249
Adolescent gender	0.482	0.663	0.060	− 0.828	1.792	0.727	0.468
Family income	− 0.117	0.444	− 0.024	− 0.995	0.761	− 0.263	0.793
Adolescent age	− 0.158	0.183	− 0.071	− 0.519	0.204	− 0.861	0.391
Adolescent race/ethnicity	− 0.473	0.701	− 0.059	− 1.860	0.913	− 0.675	0.501
TAU	− 0.672	0.776	− 0.085	− 2.206	0.862	− 0.866	0.388
TAU+	0.588	0.773	0.073	− 0.940	2.115	0.760	0.448
Task agreement—clinician report	0.915	0.350	0.222	0.224	1.607	2.618	0.010*

CGI-S = Clinical Global Impression-Severity scale, TAU = Treatment as usual, TAU+ = Treatment as usual plus measurement-based care. Covariates in the model were race/ethnicity, family income, gender, age, and treatment condition. For race/ethnicity, non-Hispanic White was used as the reference category and coded as 1, other variables = 0. For the gender variable, male was the reference category and coded as 1 and female/other was coded as 0. Holms corrected *p < 0.025

The model containing adolescent-reported task agreement as a predictor of therapy compliance was significant (*F* [1, 121] = 7.660, *p* < 0.001), and the individual adolescent report variable emerged as a significant predictor (*B* = 0.294, *SE* = 0.087, *p* = 0.001). Adolescent-reported task agreement explained 6.2% (*R*^2^) of the variance in therapy compliance. Table 11 contains full results.

**Table 11:** Regression analysis of task agreement—adolescent report on clinician rated compliance

Predictors	Unstandardized coefficients				Standardized coefficients		95.0% confidence interval for B				*t*		Sig
	B		Std. error		Beta		Lower bound		Upper bound				
(Constant)	1.607		1.045				− 0.462		3.676		1.538		0.127
CGI-S	− 0.171		0.108		− 0.123		− 0.385		0.042		− 1.588		0.115
Adolescent gender	− 0.454		0.179		− 0.192		− 0.809		− 0.099		− 2.534		0.013
Family income	0.165		0.121		0.115		− 0.075		0.405		1.361		0.176
Adolescent age	0.187		0.052		0.280		0.084		0.290		3.604		< 0.001*
Adolescent race/ethnicity	0.250		0.195		0.104		− 0.137		0.637		1.279		0.203
TAU	0.682		0.207		0.286		0.272		1.092		3.293		0.001*
TAU+	0.855		0.210		0.357		0.440		1.270		4.077		< 0.001*
Task agreement—adolescent report	0.294		0.087		0.256		0.121		0.467		3.360		0.001*

CGI-S = Clinical Global Impression-Severity scale, TAU = Treatment as usual, TAU+ = Treatment as usual plus measurement-based care. Covariates in the model were race/ethnicity, family income, gender, age, and treatment condition. For race/ethnicity, non-Hispanic White was used as the reference category and coded as 1, other variables = 0. For the gender variable, male was the reference category and coded as 1 and female/other was coded as 0. Holms corrected *p < 0.013

The overall model testing clinician report of task agreement on therapy compliance was statistically significant (*F* [1, 142] = 21.680, *p* < 0.001) and the individual clinician report variable was also a significant predictor of increased therapy compliance (*B* = 0.660, *SE* = 0.075, *p* < 0.001). Clinician-reported task agreement explained 24.4% (*R*^2^) of the variance (see Table 12).

**Table 12:** Regression analysis of task agreement-clinician report on clinician rated compliance

Predictors	Unstandardized coefficients		Standardized coefficients	95.0% confidence interval for B		*t*	Sig
	B	Std. error	Beta	Lower bound	Upper bound		
(Constant)	1.300	0.778		− 0.237	2.837	1.672	0.097
CGI-S	− 0.154	0.085	− 0.104	− 0.323	0.015	− 1.801	0.074
Adolescent gender	− 0.458	0.143	− 0.184	− 0.740	− 0.176	− 3.209	0.002*
Family income	0.035	0.096	0.023	− 0.154	0.224	0.367	0.714
Adolescent age	0.128	0.039	0.187	0.050	0.206	3.246	0.001*
Adolescent race/ethnicity	0.219	0.151	0.088	− 0.080	0.517	1.449	0.149
TAU	0.440	0.167	0.180	0.110	0.771	2.635	0.009*
TAU +	0.893	0.166	0.360	0.564	1.222	5.366	< 0.001*
Task agreement—clinician report	0.660	0.075	0.517	0.512	0.809	8.770	< 0.001*

CGI-S = Clinical Global Impression-Severity scale, TAU = Treatment as usual, TAU+ = Treatment as usual plus measurement-based care. Covariates in the model were race/ethnicity, family income, gender, age, and treatment condition. For race/ethnicity, non-Hispanic White was used as the reference category and coded as 1, other variables = 0. For the gender variable, male was the reference category and coded as 1 and female/other was coded as 0. Holms corrected *p* < 0.017

### Post hoc results

In the post hoc regression model that examined adolescent report of goal consensus and task agreement together as predictors of therapy attendance, the overall model was statistically significant (*F* [2, 120] = 2.455, *p* = 0.013), but neither adolescent report of goal consensus (*B* = 0.953, *SE* = 0.634, *p* = 0.531) nor task agreement (*B* = 0.402, *SE* = 0.640, *p* = 0.135) emerged as significant individual predictors of number of therapy sessions attended.

In the post hoc regression model that examined adolescent report of goal consensus and task agreement together as predictors of therapy compliance, the overall model was statistically significant (*F* [2, 120] = 7.818, *p* < 0.001), but only adolescent report of goal consensus (*B* = 0.424, *SE* = 0.618, *p* = 0.013) emerged as a significant individual predictor of therapy compliance, while task agreement (*B* = 0.075, *SE* = 0.169, *p* = 0.658) did not.

In the post hoc regression model that examined clinician report of goal consensus and task agreement together as predictors of therapy attendance, the overall model was not statistically significant (*F* [2, 141] = 1.215, *p* = 0.291), and neither clinician report of goal consensus (*B* = 0.049, *SE* = 0.663, *p* = 0.941) nor task agreement (*B* = 0.872, *SE* = 0.680, *p* = 0.202) were significant individual predictors of number of therapy sessions attended.

In the post hoc regression model that examined clinician report of goal consensus and task agreement together as predictors of therapy compliance, the overall model was statistically significant (*F* [2, 141] = 19.340, *p* < 0.001), and clinician report of task agreement (*B* = 0.538, *SE* = 0.146, *p* < 0.001) was as a significant individual predictor of therapy compliance, while goal consensus (*B* = 0.140, *SE* = 0.142, *p* = 0.327) was not.

There were no concerns with multicollinearity within these models, with VIF ranging from 3.90 to 4.16. These findings indicated that, when run in multi-predictor models by reporter, adolescent report of goal consensus predicted therapy compliance, and clinician report of task agreement significantly predicted therapy compliance. In these post-hoc analyses, neither goal consensus nor task agreement were significant predictors of therapy attendance.

## Discussion

Treating emotional problems in adolescents is crucial for improving functioning and well-being. Research in youth populations has demonstrated that higher attendance and greater compliance improve treatment outcomes [42]. Thus, identifying factors that reduce attrition and improve compliance in therapy hold promise for improving treatment outcomes. Toward that end, this study examined two unique components of the working alliance, namely goal consensus and task agreement as predictors of therapy attendance and compliance as these factors have been understudied, especially in adolescent populations. Overall, findings from the current study supported our hypotheses, indicating that the more adolescents and clinicians perceive they are collaborating on setting goals, working mutually toward achieving these goals, and are clear about how to make that change happen, the more adolescents attend therapy sessions and comply with assigned treatment tasks. This is consistent with other studies that have examined working alliance more broadly in youth populations [11, 14, 41].

### Goal consensus

As noted, the results indicated that higher goal consensus predicted both higher therapy attendance and therapy compliance, from both a clinician and adolescent perspective. These findings have several interpretations and implications for the treatment of adolescent emotional problems. Specifically, the findings suggest that the process of setting and agreeing on goals could be motivating for treatment. For example, prior research on SMART (specific, measurable, achievable, realistic, and timed) goal setting [7], a multidimensional construct designed to create a motivational framework for setting goals, indicates that collaborating on goals may help bolster attendance and compliance. Emphasizing collaborative goal setting can also inform how clinicians in the community may leverage goal setting to improve therapy session attendance and compliance. Specifically, clinicians providing more collaboration and structure while setting goals by mutually specifying the target activity and support needed, quantifying the performance, and agreeing on the time to achieve the desired state might enhance attendance and treatment compliance. When clinicians and adolescents agree on their therapy goals, they may be touching on these dimensions, leading to greater motivation for change in the adolescent and therefore greater treatment attendance and compliance. Findings of post hoc analyses indicated that adolescent perspective on therapy goal consensus may impact therapy compliance more than perceptions of task agreement, meaning that the extent to which adolescents believe that they and their clinician agree on the goals of therapy might better lead to higher compliance with therapy than adolescent perspective on their and their clinician’s agreement on the steps needed to reach therapy goals. This further indicates that clinician and adolescent agreement on therapy goals is important for improving therapy compliance and, therefore, therapy outcomes.

### Task agreement

Results of this study indicated that higher adolescent-clinician agreement on therapy tasks predicted higher therapy compliance and attendance. These findings contrast with Hagen et al. [24], who found that adult clients report of task agreement was not a predictor of treatment outcomes and instead suggest adolescents’ “buy in” may be particularly important to improving therapy compliance and attendance. The current findings suggest that agreeing on specific therapy tasks needed to achieve therapy goals is important and likely to enhance both therapy attendance and compliance. This process likely requires consistent and quality communication. In contrast, when a perceived mismatch occurs between client and clinician on the specific therapy tasks needed to achieve goals, it could be representative of broader poor communication, a topic addressed in a recent review of adolescent working alliance research [12]. In this review, Cirasola et al. [12] identified alliance strains as a factor that reduces adolescent compliance. Based on the current post hoc findings, clinician’s perspective on task agreement, which might include regular check-ins with adolescent clients about the steps they need to take to reach their goals, and being aware of potential alliance ruptures, could enhance compliance [12].

While both goal consensus and task agreement were significant predictors of therapy attendance and compliance, clinician perspective of task agreement notably accounted for nearly one quarter of the variability of therapy compliance, a relatively high proportion of variance for clinical medicine research. The importance of clinician perspective of task agreement in predicting therapy compliance was further supported by the findings of the more conservative post hoc models, in which clinician report of task agreement remained a predictor of therapy compliance, the amount of variability of therapy compliance accounted for by these variables ranged from 6.2 to 24.4%, while in the attendance analyses, they accounted for between 3.4 and 12.8%. These findings may indicate that therapy attendance is affected by more external or contextual factors, whereas therapy compliance is impacted by factors specific to the clinician and adolescent, including goal consensus and task agreement. It is clear that these studied factors are not the only drivers of treatment attendance and compliance. Other factors that may lead to low levels of attendance and compliance include clinician-level factors, such as clinicians being too focused on delivering the specifics of their intervention and neglecting consensus building, adolescent/family specific barriers such as attitudes about therapy and external factors such as transportation, or financial stressors.

While higher goal consensus and task agreement were found to predict higher attendance and compliance, the directionality of the relationship between goal consensus, task agreement, therapy attendance, and compliance is not completely clear. It is possible that attending a higher number of therapy sessions further develops the working alliance and therefore bolsters attendance and compliance. Additionally, it may be possible that the relation between goal consensus, task agreement, attendance, and compliance is transactional, with both factors influencing each other at different stages of the therapeutic process. Other variables, like personality, could also be influencing this relationship. For example, an adolescent’s personality could be responsible for better treatment compliance rather than the goal consensus or task agreement itself. Adolescents that tend to be more agreeable, conscientious, or compliant may also be good at agreeing on goals with their clinicians as a byproduct.

Interestingly, the associations between adolescent and clinician report of goal consensus and task agreement were statistically significant but the magnitude was low. This is consistent with other working alliance research that has found the correlation between clinician-client ratings of the working alliance to be low [32, 38]. This would indicate that perceptions from both parties are valuable and articulated in therapy by actively and continuously checking in about goals explicitly throughout the treatment process.

## Limitations and future directions

The current study has several limitations. A single item measure was used for compliance instead of a multi-item measure. Related, measures of compliance that are linked to specific treatment method, such as a compliance measure for CBT or IPT, may provide a more comprehensive assessment of compliance. A multi-item measure of compliance could evaluate differences in compliance within sessions, between sessions, and also better assess for homework completion. Additionally, measures of compliance that are unique to a primary client diagnosis may improve understanding of predictors of compliance [51]. In the current study, therapy compliance was clinician-rated, but more insight may be attained by incorporating other perspectives, such as observer, parent, or youth rated reports of compliance.

The two components of working alliance included in the current study, goal consensus and task agreement, were highly correlated in the current sample, which may indicate that they are measuring highly related constructs. In fact, some research supports that working alliance has better model fit with two structures [18]. Future researchers would be well served to assess the psychometric reliability and validity of these three aspects of working alliance, as well as examine the factor analysis of the construct. This would be particularly important to examine in youth community samples, as the vast majority of studies using this measure have adult samples [5].

In addition to incorporating additional perspectives of compliance, parent and observer-rated measures of working alliance exist but were not included in the current study. The working alliance measure used in the current study, the Working Alliance Inventory, has not been validated for parent-report. However, the parent perspective on working alliance would also be important to evaluate as parent involvement in the therapeutic process for youth is vital and often improves youth outcomes [40]. Future researchers examining adolescent-clinician working alliance may also benefit from incorporating observational ratings. For example, the Therapy Process Observational Coding System—Alliance scale (TPOCS–A), is an observational measure that was developed for adolescent populations and can be used to assess the working alliance between the child and clinician or parent and clinician [36]. The TPOCS-A has been found to predict improved therapy outcomes for youth with internalizing disorders in community mental health clinics. While the current study used quantitative methodology to examine the relation between goal consensus, task agreement, and compliance, qualitative methods or mixed methods approaches could shed more light on how adolescents view the importance of agreeing on therapy goals and tasks which could potentially lead to the identification of ways to improve goal setting in the treatment process.

While the current study used data on goal consensus and task agreement at one time point, measuring these constructs at each therapy session could provide information about the stability of goal consensus and task agreement, while also potentially identifying any critical points in the treatment process where these constructs should be emphasized. Future research could also examine whether types of goals affect attendance or compliance. For example, a therapy goal may include short-term goals like conversing with an unfamiliar peer, to long-term goals like developing a new friend group. Types of goals may be influenced by the treatment approach, the setting, population, client primary diagnosis and therefore might be informative to examine and integrate into analyses relating to goal consensus.

Another factor that might limit generalizability is that the clinicians were volunteers or novices in some of the treatments and most had master’s rather than doctoral degrees. Additionally, adolescents and clinicians who volunteer to participate in a randomized controlled trial may not reflect those working or seeking services in the general population. In the current sample, the majority of participants were female and white, and adolescents were skewed to low income which might limit generalizability of the current findings.

Finally, the use of attendance as a measure of “successful outcome” may not represent a better treatment response. Namely, low number of sessions attended may reflect poorer or better outcomes. In addition, other relevant factors that can contribute to treatment attendance and compliance (i.e., financial constraints, transportation difficulties, stigma around mental health) were not fully accounted for in the current study. Results of the post hoc analyses did not find goal consensus or task agreement to predict attendance. Therefore, the current predictors of attendance should be interpreted with caution and further research should examine other factors that may lead to better therapy attendance.

## Clinical considerations

The current findings highlight important considerations for clinicians working with youth with emotional disorders in community settings. Given the impact of goal consensus and task agreement on therapy attendance and compliance in the current sample, there appear to be benefits for clinicians to take the time during each therapy session to ensure that they discuss and agree with youth on the specific goals of treatment and the steps that would get youth toward those goals. The mental health field is moving toward adoption of a measurement-based care model, where continuous youth-report data is used to support clinical decision making. This methodology has been shown to improve youth-clinician alliance and clinical outcomes [35]. For example, clinicians can have youth rate the working alliance each session using a measure like the Session Rating Scale, which is designed to assess for each component of the working alliance at every session [16]. Collecting session-by-session ratings of working alliance would allow clinicians to track changes in therapeutic bond, goal consensus, and task agreement over time and identify any potential opportunities to address concerns. In doing so, they may be able to increase youth therapy attendance and treatment compliance and ultimately improve treatment outcomes for youth with emotional disorders.

## Conclusions

Adolescents with emotional disorders are more likely to attend and comply with treatment when they and their community-based clinicians perceive they agree on therapy goals and tasks together. These findings suggest that to bolster attendance and improve therapy compliance, clinicians should focus on communicating with adolescents around setting goals and the steps needed to achieve those goals. This is particularly relevant for treatments delivered in community mental health centers given the high rates of attrition [20]. Future research should examine goal consensus and task agreement at multiple points in the treatment process and incorporate multiple reporters of compliance and working alliance. Additional factors, beyond the working alliance would also be important to identify as a means of improving attendance, compliance, and the quality of care for adolescents struggling with emotional disorders who are served in community mental health centers.

## Data Availability

The datasets generated and/or analyzed during the current study will be made publicly available once the primary outcomes paper is accepted for publication and are available from the corresponding author on request.

## Abbreviations

SMART: Specific, measurable, achievable, realistic, timed
COMET: Community study of outcome monitoring for emotional disorders in Teens
NIMH: National Institute of Mental Health
CBT: Cognitive behavioral therapy
YOQ: Youth outcomes questionnaire
IPT: Interpersonal therapy
CBT: Cognitive behavioral therapy
ADIS: Anxiety disorders interview schedule for the DSM-5
